# Supplementary material for: On the evaluation of research software: the CDUR procedure
Source: F1000Res. 2019 Nov 26;8:1353. Originally published 2019 Aug 5. [Version 2] doi: 10.12688/f1000research.19994.2 (PMC6883399; doi:10.12688/f1000research.19994.2)
Supplement: Peer review history

# Peer review: Reviewer response for version 2

**DOI:** 10.5256/f1000research.23236.r57114

**Published:** 2019-11-27

**Authors:** Francisco Queiroz

**Affiliations:** 1 School of Design, University of Leeds, Leeds, UK

The authors have provided a revised article addressing the most important issues identified by the previous report. Overall, the improvement of Figure 1,  the inclusion of the use case, and the call to action have made the article much more comprehensible and engaging – in which case I'd recommend its approval without any reservations.

I confirm that I have read this submission and believe that I have an appropriate level of expertise to confirm that it is of an acceptable scientific standard.

# Peer review: Reviewer response for version 1

**DOI:** 10.5256/f1000research.21946.r52525

**Published:** 2019-09-23

**Authors:** Jean-Pierre Merlet

**Affiliations:** 1 HEPHAISTOS Project, Inria, Inria, Sophia-Antipolis, Valbonne, France

This paper gives a good definition of what is a research software (RS) and I agree with the authors that an extensive version of what is a RS. I have however various remarks:

- A career of a researcher has extensively changed in the recent years: more chairs available for a relatively short period of time (typically 5 years) are given so that in this limited amount of time a relatively young researcher cannot afford to stand by the dissemination and maintenance work required by the evaluation scheme provided by the authors
- The authors still favor a specific type of RS, with availability, documented, licensed and with versioning. This is dangerous as many RS do not enter in this category: software that is produced to check a specific scientific result, software specifically designed to manage a particular system etc.. These software are not intended to be disseminated either as they are used only for checking and benchmarking or because they are very specific (for example a real-time software where the purpose is to save as much as possible computation time so that it cannot be general by essence as any general procedure will require tests that will penalize the performances) and therefore cannot be reproducible. As developing software is time intensive having too strict rule for the evaluation of software (such as documentation and availability) may discourage researchers producing them  although they provide some good idea of the efficiency of the underlying theoretical work.
- Citations of publications as a measure of impact is already questionable and I will say this is even worse for RS. It is quite current in the computer science community to say that the only good software is the one produced by the authors so that citations is limited and usually not very positive.
- I fully agree that software development may be a major component in the daily activity of a researcher and therefore must be taken into account in our evaluation. However the specificities of the RS and of the domain must also be taken into account in this evaluation. The CDUR procedure proposed by the authors is very fine but can be applied only on a limited number of RS types and his application will be disastrous for other types. For example it is easy to find RS for which the CDU part is not relevant while the R part is the only one of importance: for example a RS that performs the control of a specific robotic system cannot be reproducible (unless the reviewer has at hand an exactly similar robot), its use is limited to a specific prototype (and the RS has been designed for it) and has to bey tailored to manage another system so it cannot be disseminated. Hence the only evaluation criteria is the R part which is illustrated by the performance of the whole system, being given the performance of the hardware.
- I recognize that the authors are right in term of objectives: our current approach of RS development may lead to a waste of time with researchers programming again and again the same algorithms instead on focusing on their specific objectives. Some kind of mutualization will be beneficial provided that he RS is sufficiently disseminated and open to the community. But a researcher has to find the right balance between his/her research activity and the time devoted to maintenance, documentation and dissemination, a balance that greatly depends upon the domain. Furthermore good software practices for development is also dependent of the domains.

In summary although the authors have been very careful in their definition and evaluation rules and the absolute necessity of flexibility in the evaluation rules, they ended up with an evaluation procedure that may lead researcher to avoid going into the business of RS development. But this is nice paper that is worth being indexed for opening a discussion on RS evaluation according to the specific domain and context in which it has been developed.

I confirm that I have read this submission and believe that I have an appropriate level of expertise to confirm that it is of an acceptable scientific standard.

# Peer review: Reviewer response for version 1

**DOI:** 10.5256/f1000research.21946.r53711

**Published:** 2019-09-13

**Authors:** Francisco Queiroz

**Affiliations:** 1 School of Design, University of Leeds, Leeds, UK

Overall, this is a clear, well-written article outlining a novel procedure that could impact, in positive ways, policies and practices regarding scientific software development and impact. However, I believe there are some aspects of the article that could be either improved or clarified.

1. The study seems to be mostly based on secondary research – more specifically, arguments and ideas are developed based on findings from literature. Moreover, it can be assumed, the study is informed by the proximity of the authors to the academic/professional environment it investigates. However, there is no clear indication on how the material that serves as basis for the analysis was selected (indexed databases? specialized resources such as publications and conferences?), in which case it would be interesting to include a justification on why those sources are relevant.
2. Additional material that could, potentially, be referenced are Chue Hong`s position paper on the need for a framework for comparing software ^1^; and also ESRC's plans of developing a software accreditation framework ^2^.
3. In section 2, the authors write: "Note that to cite software in science is different to citing scientific (or more precisely, research) software, which is the issue here". Perhaps the difference could be clarified?
4. In 2.1, the authors write: "in 12,13 we can find the following definition". Given that the definition`s authorship is originally from 12, it would be probably more appropriate to write something along the lines of "a definition elaborated by 12 was summarized by 13 as" 
5. There are several passages in both French and English. Wouldn't it be enough to keep the English versions (along with an indication that they were translated from French by the authors?)
6. The authors write: "Finally, let us mention that, as in the case of publications, the research software production of a laboratory is decided and proposed by the lab’s members, and it is approved by the leading institutions during the usual laboratory evaluation and funding procedures". Is that true for every case of research software production and are there any references supporting that? 
7. Figure 1, illustrating concepts appearing in the study, could be improved, possibly by matching label colors to the colors of the rectangles for easier identification.
8. Is not clear why there are two screenshots of Plume (one in French and one in English). One would probably be enough?
9. The authors write: "(...) we remark that there can be several classic articles associated to a single RS, and that could be used as its reference". Could references to some of those classic articles be provided?
10. The study ends without (i) considerations on future research, (ii) clear indications on initiatives/pilots for the procedure, (iii) a call to action for the community to test it. Overall, it's not clear what the next steps are.
11. Regarding the applicability of the procedure, the article would benefit from including a table or diagram (probably in Section 4) explaining the procedure, preferably featuring a clear, step-by-step description of the process. That should clarify how CDUR could be adopted, increasing the potential for its adoption and use.   

I confirm that I have read this submission and believe that I have an appropriate level of expertise to confirm that it is of an acceptable scientific standard, however I have significant reservations, as outlined above.

## References

[1] : Why do we need to compare research software, and how should we do it? in Proceedings of the Fourth Workshop on Sustainable Software for Science: Practice and Experiences (WSSSPE4). *University of Manchester, CEUR Workshop Proceedings*.2016;

[2] EPSRC Software Infrastructure strategy 2018. *EPSRC*.2018; Reference source
